# Understanding the impact of the COVID-19 pandemic on psychiatric trainees and what can help

**DOI:** 10.1192/bjb.2022.33

**Published:** 2023-08

**Authors:** Matthew Anastasis, Robert Freudenthal, Jo O'Reilly

**Affiliations:** 1 Barnet, Enfield and Haringey Mental Health NHS Trust, UK; 2 Camden and Islington NHS Foundation Trust, UK

**Keywords:** Coronavirus, COVID-19, burnout, well-being, trainees

## Abstract

The COVID-19 pandemic has increased rates of psychological distress and burnout in healthcare staff. How can we understand our experiences of the pandemic? We reflect on the experiences of psychiatry trainees in two north London mental health trusts. From a psychoanalytic understanding, states of extreme anxiety can lead to a manic defence and functioning in the paranoid–schizoid position. This position is derived from object relations theory and is characterised by binary thinking, splitting, projection, defensiveness and ‘knee-jerk’ decision-making. This can affect our perceptions, responses to others, relationships and ability to function and, therefore, our clinical practice and well-being. We consider the importance of recognising these processes and of organisational containment and having space to reflect. This supports functioning in the depressive position, a state of mind where we can tolerate anxiety, address difficult realities and develop new ideas. We hope these understandings are helpful to our colleagues in all professions.

The coronavirus disease 2019 (COVID-19) pandemic has dramatically changed the way we all work in healthcare, including within mental health services. The impact of the pandemic on staff has been considerable. In 2020, burnout was one of the most common reasons for psychiatrists contacting the Psychiatrists’ Support Service run by the Royal College of Psychiatrists.^1^ More widely, half of the doctors who responded to a survey by the British Medical Association considered that they were currently suffering from depression, anxiety, stress, burnout, emotional distress or another mental health condition, and this was worse in three-quarters of respondents than before the pandemic.^2^ Over 90% of nurses said they were more anxious than before the pandemic in a survey by *Nursing Times*.^3^ An increased rate of burnout during the pandemic has also been shown worldwide.^4^ A survey of psychiatrists in India showed that the majority of respondents were worried about various issues including working in a hospital, getting infected and the health of elders in the family.^5^ Interestingly, however, studies have shown mixed responses. Some studies showed that medical staff working on the COVID-19 front line had a lower frequency of burnout compared with those working on their usual wards.^4^ Rates of burnout during the pandemic were lower than expected in psychiatry trainees according to a survey in Saudi Arabia.^6^

The impact of the pandemic on staff is important to consider as it not only affects staff well-being but can affect the care that services provide to patients. The impact on trainees should also be highlighted, as younger and more junior doctors are at greater risk of burnout and psychological distress.^6–9^ This can affect trainee retention. But how can we understand our response to the pandemic and what can help?

In this paper, we reflect on the impact of the COVID-19 pandemic on psychiatry trainees. Reflections stem from the personal experiences of two authors of this paper (M.A. and R.F.), who have had first-hand experience of working as junior and senior psychiatry trainees during the first wave of the pandemic and were trainee representatives for both core and higher psychiatry trainees. Reflections also stem from feedback sought from the wider trainee body of junior and senior trainees working across two mental health trusts in north London. We sought the opinion of trainees with varied experiences, wherever their place of work, including those who were shielding. We presented these experiences at a forum in an academic programme attended by over 100 doctors from the two mental health trusts; then, we facilitated a discussion of trainees’ experiences that provided further insight.

Here, we consider three themes that have arisen from the above reflections on psychiatry trainees’ experiences of the pandemic: (a) anxiety and the workplace; (b) containment and the workplace; and (c) the need to reflect. For each theme, we consider models that can help understand these responses and the response of the organisation, and what may help in the future.

## Anxiety and the workplace

### Experiences

A common theme that arose among psychiatry trainees in their feedback was the anxiety and uncertainty that they experienced during the pandemic. This uncertainty affected both their training and jobs. Would they be moved from their current jobs to the acute wards? This did happen for a number of trainees. The psychiatric ward became a more chaotic environment. The risk assessment of patients took on a new dimension owing to the risk of catching COVID-19 on the ward. Therefore, the threshold for admission increased, and the severity and acuity of patients’ presentations seemed to escalate. Quite understandably, patients who were very psychiatrically unwell often did not follow COVID-19 isolation procedures, which made the situation feel less contained. Disinhibited patients coughing and being unable to keep distance, followed by spiking temperatures, occurred with some frequency. New psychiatry trainees in particular found working in this environment daunting, and the atmosphere was described as unremittingly ‘tense’. Trainees spoke of their worry about catching the virus at work and many did become unwell. Unfortunately, there were reports of junior doctors being physically attacked in these new working environments. There was a sense that junior doctors had to ‘step up’ and rise to the challenge, get ‘stuck in’ and be – for want of a better phrase – strong and stable, and adaptable to rapidly changing new environments, despite being put in actual physical and psychological harm's way. One trainee encapsulated these experiences thus: ‘I was on an acute ward. When it was understaffed we had difficulties containing patients, which meant I was physically assaulted twice by the same patient. This, added to the general acuity, stress and long hours meant it was some of the most stressful and daunting times of my entire working life.’

There were also opportunities. In their role as junior doctors, they found ways to manage these extraordinary circumstances using their knowledge and expertise. There were examples of doctors leading on innovative new practice, for example, sourcing cannulas, intravenous fluids and oxygen cylinders for patients on the old age psychiatric ward to prevent them being transferred to the accident and emergency department. For some, the experience felt exciting, with a sense of elation and a belief in their ability to manage and cope with the additional stress. This, however, was difficult to sustain over a long period of time, and potentially led to exhaustion and burnout.

### Understanding and working through these experiences

In order to understand these experiences, we must understand the effects of anxiety. In comparison to the virus, anxiety and fear can permeate everywhere, penetrate our defences, and make us react and behave irrationally. Anxiety in the workplace is inevitable and often helpful – in motivating us to take necessary actions. However, fear and excessive anxiety affect our ability to work.

Increased anxiety brings increased defences against anxiety. These may become problematic if they become excessive or rigid and distort the reality of the actual danger and our own vulnerability. Excessive anxiety can paralyse us and lead to avoidance and withdrawal, with accompanying feelings of guilt and shame. Conditions of fear and loss may also activate a manic defence. This is characterised by omnipotence, a denial of vulnerability and even elation. This can lead us to place ourselves at increased risk by behaving as if we are invincible (like putting ourselves in actual harm's way and at risk of assault) and by not attending to our own well-being. This may help explain the sense of excitement and elation experienced by some trainees and why there was less burnout than expected in some studies in front-line workers in the early stages of the pandemic.

Excessive anxiety and fear also lead to states of mind that aim to ensure survival against threat – a ‘fight or flight’ mode. Ideas from psychoanalysis about early psychic life and psychological functioning under conditions of threat can be helpful in understanding some of the processes that may become activated by perceived danger, both individually and organisationally. Melanie Klein described ‘paranoid–schizoid’ states of mind, characterised by binary thinking and splitting of experiences into good and bad, projection of unacceptable thoughts and feelings into others, idealisation and denigration, and blame and accusation.^10^ These states of mind can provide certainty and simplicity to a mind (and an organisation) under threat. However, it can also mean that we feel defensive, persecuted and overly responsible for all that happens at work, a state of mind that makes it difficult to listen, to process information and to think. This may lead to action being taken in a ‘knee-jerk’ manner. This can challenge team cohesion and increase conflict throughout the organisation. These states of mind can also be difficult to get out of and can lead to isolation and exhaustion.

Processing and coming to terms with loss and threat are hallmarks of functioning in the depressive position, a more mature reality-based state of mind according to Melanie Klein's description of early psychic development.^11^ It is also a helpful model in thinking about working in mental health services during the pandemic; in the depressive position, we are better able to tolerate anxiety, can bear difficult realities, are better able to think about complex issues, can address the reality of loss and uncertainty, and can find solutions. There is less projection into others, and we are better able to work alongside anxiety. Recognition of our own vulnerability and limits supports us to prioritise our own needs and safety in a realistic manner, and we are less likely to behave as if we are invincible. Mourning the reality of what has been lost allows reparative measures to be put in place where possible, and for grief to be processed.

We can all oscillate between paranoid–schizoid and depressive positions, depending on the level of threat and our own individual situations. From the perspective of the mental health organisation, activities that support staff to reflect and to face painful realities in their work enhance the capacity of the organisation to contain anxiety and to provide services based on understanding and thought to their patients.

## Containment and the workplace

### Experiences

The experience of working out of hours was another theme that arose for trainees. Communication with the medical teams was more difficult, and there was a feeling of not wanting to bother them with phone calls. One trainee remarked that they were worried their ‘GMC registration was truly on the line’. Language used in the media made reference to a ‘tsunami’ or ‘wave’ of mental health difficulties approaching. Being the on-call psychiatrist working alone overnight, expecting to face this tsunami, elicited feelings of dread. As one trainee put it: ‘*being the only doctor on site was quite daunting at the peak … I felt like I would have rather been on a medical ward where at least there was a team around you.*’

Furthermore, their contact and proximity to each other and to their patients was perceived as a source of danger. Some found it disturbing that the concept of trainees sitting together in a room suddenly shifted from a potential source of support to being a threat. For some new trainees, it was particularly difficult to feel part of a cohesive ‘trainee body’ when they had met very few of the other trainees in person. Some trainees had to shield, which made their vulnerability, or the vulnerability of those close to them, explicit to their colleagues. This public revelation of personal vulnerability could feel exposing. People working at home were particularly disconnected. The usual support structure of contact with the team and colleagues was lost. This also could lead to feelings of guilt at not being on the ‘front line’ like their colleagues.

### Understanding and working through these experiences

In psychiatry, our daily work requires us to be in contact with, contain, understand and manage extreme states of disturbance and distress. Psychiatry trainees, in particular, work at the ‘coalface’ of human distress and are often the first port of call in addressing distress and disturbed mental states. In order to do this work, trainees themselves need to be supported and their anxieties contained. The psychiatrist and psychoanalyst Bion referred to this with his concept of the ‘container contained’.^12^ In an organisational sense, containment for staff is usually provided through a hierarchical framework of activities that provide space to think in a multi-layered way. Activities include formal meetings, supervision, Balint groups, reflective practice and specialist panels that consider high-risk or complex patients. It also includes our informal contacts and conversations with each other. These are hugely important in sharing dilemmas and anxieties, creating ideas, finding solutions and taking pleasure in our work. Our relationships with each other are the bedrock that determines how much we thrive or struggle at work. Containment then is found in a relational context and is embedded in the fabric of the mental health organisation in ordinary times.

The loss of usual services and training activities for trainees, and the loss of the physical workplace for some, has become the everyday reality at work. We were asked to see ourselves as potential vectors of infection, which led to the loss of close contact with colleagues that might sustain and contain us. Remote working, physical distancing and wearing masks underscore this. The capacity to contain ourselves, our colleagues and our patients has been under intense pressure.

## The need to reflect

### Experiences

Some trainees expressed relief and were thankful at being asked about their experiences during the pandemic. It was as if the impact on them had not been shared and considered. This was displayed during an afternoon of the academic programme that was devoted to trainees’ experiences of the pandemic, where an impassioned discussion developed which could have continued long after the scheduled end of the programme.

Trainees also expressed gratitude to seniors: ‘*the site tutors made a fantastic effort to keep morale up and have us meet when we could. They also got the academic programme back up and running remotely which was appreciated.*’ Another trainee fed back: ‘*I think we were very lucky … having so many decent, warm and importantly available seniors.*’

Some trainees found it helpful to reflect on the ethical dilemmas that arose. There was concern about the impact of the restrictions on their patients, with a sense that the realities of people with severe mental illness were being forgotten about in the mainstream reporting. There were questions about whether it was appropriate to remove facemasks if it was felt that they were interfering in developing a therapeutic relationship between staff and patients. There were particular legal concerns, for instance, higher trainees wished to ensure that the Mental Health Act was not inappropriately used to ensure that patients remained isolated.

### Understanding and working through these experiences

The pandemic exposed our own personal relationship with risk and liberty; therefore, some felt able to learn about themselves through the crisis. Their recognition of this is suggested by trainees’ relief at being asked about their experiences of the pandemic, the helpfulness of Balint groups, the lively discussion of their experiences that arose in the forum in the academic programme and other opportunities to process the emotional impact.

The trainees also felt supported by the medical leadership through their availability and their support in continuing activities that allowed trainees to reflect, such as the academic programme and Balint groups. Importantly, these activities showed that the senior staff were not overwhelmed and were future-oriented.

These experiences highlight the importance of reflection to address the emotional and psychological impact of our experiences at work as a basis for developing new thoughts and ideas and addressing difficult realities.

## Looking forward

We are now in a new phase of the pandemic. The vaccine has helped to alter the course of the pandemic and reduced the threat of the virus for most people. However, the emergence of new variants, such as the omicron variant, has shown that the trajectory of the pandemic is far from certain, and we will continue to face new challenges. Furthermore, usual services are now resuming in the context of learning to ‘live with the virus’. Thus, people are being encouraged to be in closer contact with each other again. This can affect both staff and patients differently, and new anxieties, vulnerabilities and dilemmas are likely to continue to emerge. Things may never go back to ‘normal’, as we understood it before the pandemic. Clinicians are facing a backlog of clinical work in the National Health Service and there is pressure to tackle this. Therefore, we must use the opportunity to process and understand our experiences. Otherwise, the prospect of a protracted pandemic and adjustment to a new normal after the pandemic will inevitably lead to feelings of dread, exhaustion and burnout. Reflecting on what we have experienced and how it has made us feel is the first step. The psychiatry trainees’ experiences have highlighted the importance of addressing the emotional and psychological impact of experiences at work, being supported and contained by both senior colleagues and by the organisation, and having space to reflect. This will help clinicians, including trainees, to be in a position to address and work through difficult realities and develop new thoughts and ideas. In psychiatry, we are particularly fortunate to have the opportunity to consider the psychodynamic factors in our work and within the organisation. We hope that the understandings discussed in this paper will be helpful to our colleagues of all professions and in all specialties.

## About the authors

**Matthew Anastasis** is a core psychiatry trainee at Barnet, Enfield and Haringey Mental Health NHS Trust, London, UK; **Robert Freudenthal** is a higher psychiatry trainee at Barnet, Enfield and Haringey Mental Health NHS Trust, London, UK; **Jo O'Reilly** is a psychiatrist and medical psychotherapist at Camden and Islington Psychodynamic Psychotherapy Service, Camden and Islington NHS Foundation Trust, London UK.
